# Efficient pairwise RNA structure prediction using probabilistic alignment constraints in Dynalign

**DOI:** 10.1186/1471-2105-8-130

**Published:** 2007-04-19

**Authors:** Arif Ozgun Harmanci, Gaurav Sharma, David H Mathews

**Affiliations:** 1 Department of Electrical and Computer Engineering, University of Rochester, Hopeman 204, RC Box 270126, Rochester, NY 14627, USA; 2 Department of Biostatistics and Computational Biology, University of Rochester Medical Center, 601 Elmwood Avenue, Box 630, Rochester, NY 14642, USA; 3 Department of Biochemistry and Biophysics, University of Rochester Medical Center 601 Elmwood Avenue, Box 712, Rochester, NY 14642, USA

## Abstract

**Background:**

Joint alignment and secondary structure prediction of two RNA sequences can significantly improve the accuracy of the structural predictions. Methods addressing this problem, however, are forced to employ constraints that reduce computation by restricting the alignments and/or structures (i.e. folds) that are permissible. In this paper, a new methodology is presented for the purpose of establishing alignment constraints based on nucleotide alignment and insertion posterior probabilities. Using a hidden Markov model, posterior probabilities of alignment and insertion are computed for all possible pairings of nucleotide positions from the two sequences. These alignment and insertion posterior probabilities are additively combined to obtain probabilities of *co-incidence *for nucleotide position pairs. A suitable alignment constraint is obtained by thresholding the co-incidence probabilities. The constraint is integrated with Dynalign, a free energy minimization algorithm for joint alignment and secondary structure prediction. The resulting method is benchmarked against the previous version of Dynalign and against other programs for pairwise RNA structure prediction.

**Results:**

The proposed technique eliminates manual parameter selection in Dynalign and provides significant computational time savings in comparison to prior constraints in Dynalign while simultaneously providing a small improvement in the structural prediction accuracy. Savings are also realized in memory. In experiments over a 5S RNA dataset with average sequence length of approximately 120 nucleotides, the method reduces computation by a factor of 2. The method performs favorably in comparison to other programs for pairwise RNA structure prediction: yielding better accuracy, on average, and requiring significantly lesser computational resources.

**Conclusion:**

Probabilistic analysis can be utilized in order to automate the determination of alignment constraints for pairwise RNA structure prediction methods in a principled fashion. These constraints can reduce the computational and memory requirements of these methods while maintaining or improving their accuracy of structural prediction. This extends the practical reach of these methods to longer length sequences. The revised Dynalign code is freely available for download.

## 1 Background

With the widespread availability of data sets of genome and protein sequences, methods for analyzing the sequences to extract biologically salient information have emerged as powerful techniques in computational bioinformatics [1]. In this arena, comparative sequence analysis has proven extremely powerful, whereby sequence segments across different genomes are examined for similarities. Segments identified as similar represent evolutionarily conserved *homologs *and are deemed to be biologically significant due to their apparent preservation across the genomes. The postulated significance can then be tested with experiments, which can also help establish functional correlates. Because the biological experiments are time-consuming and expensive, the comparative analysis serves to improve efficiency by "pre-filtering" the relatively large genome to determine relatively smaller segments over which the experimental effort may be concentrated. The effectiveness of this pre-filtering step is, of course, determined by its accuracy in correctly identifying meaningful homologs and by the computational efficiency of the algorithmic implementations.

In the identification of homologous segments of genomes, sequence-alignment has been the primary workhorse since it can be directly deployed on readily available sequence data. Commonly used methods assign a score to an alignment of a pair of sequences based on nucleotide matches/mis-matches and gaps in the alignment. The similarity score for a pair of genome segments is then computed as the maximum value of this score over all potential alignments, which serves as an alignment-based measure of homology. Through algorithmic innovations (primarily dynamic programming formulations), computationally-efficient methods have been developed for sequence-alignment that can be effectively deployed over large genomes [1], The computational speed-up, however, does not ameliorate a limitation of these methods in that they rely on pure sequence-alignment whereas biological function is actually determined largely by structure, which is not necessarily in one-to-one correspondence with sequence. Structurally conserved biomolecular elements with differing sequences directly illustrate this problem. The divergence between structural and sequence homology is particularly true of non-coding RNAs (ncRNAs), where it is commonly believed that secondary structure, i.e. the sum of the canonical base pairs, is more conserved than the nucleotide sequence [2]. As a ncRNA sequence evolves, compensating changes occur that allow the sequence to drift without affecting the secondary structure. Compensating base pair changes by which secondary structure is conserved, but sequence is not, are therefore used to identify the conserved structure [3].

Given the strong correlation between structure and function observed in biological systems, it is more meaningful to explore homologies based on structure rather than sequence alone. In general, the determination of structural homology scores, i.e. a structural similarity measure for the most "similar" (or common) 3-D structure corresponding to genomic/proteomic sequence segments is a challenging problem. In the case of ncRNA, however, the problem may be rendered tractable, i.e. polynomial time complexity, by focusing one's attention on secondary structure. Sankoff [4] first proposed a dynamic programming approach to this problem that addresses a large class of secondary structures (excluding pseudoknots). Furthermore, he also illustrated how the approach can be extended to include a composite score that accounts for both sequence and structure similarity while retaining the polynomial complexity.

Though polynomial in complexity, Sankoff's proposed algorithm in its original form is still too computationally demanding for deployment on long sequences, such as 16S or 23S rRNA, in the near future (the computational complexity is *O*(*N*^6^) for two sequences of length *N*). A simplified version of the algorithm, Dynalign, implemented by Mathews et al. [5,6] constitutes one of the practical computational realizations. The method uses a heuristic to improve computational efficiency by restricting the number of possibilities examined in each dynamic programming step. However, its computational burden remains significant and no analytic guidance is available on the selection of parameter values for the heuristic simplification.

In this paper, this limitation is addressed by developing a principled mechanism for significantly improving the computational efficiency of Dynalign. The motivation for our technique arose from the "*a posteriori*" probability decoding methods developed for error correction in electrical communication systems in the early 1970s [7]. These have seen a recent re-resurgence of research interest due to the development of turbo codes [8] and re-discovery of low-density parity check codes [9] that now constitute active areas of research and development in electrical communication systems. An adoption of the *a posteriori *probability methodology allows us to determine the confidence in local accuracy of sequence alignment in a computationally efficient fashion using a Hidden Markov Model [1,10]. These probabilistic estimates of confidence in local alignment accuracy are utilized in order to define constraint windows for limiting the choices in the dynamic programming step in Dynalign. Intuitively, this process can be understood as follows: In regions with strong confidence in the alignment accuracy, the possibilities explored in the dynamic programming steps are severely restricted to options mandated by the corresponding alignment and in regions with low confidence in the alignment a wider range of possibilities are allowed in the dynamic programming steps. The method is superior to the prior heuristic of restricting the number of possibilities to an arbitrary fixed number at each step since the restrictions are based on confidence estimates in the sequence alignment and therefore cuts computation where it is not required (where the sequence similarity alone provides high confidence) and does not restrict it when it is in fact required (when sequence similarity provides little confidence). The resulting method provides a very significant decrease in the time required for Dynalign computations while simultaneously increasing (by a small margin) the accuracy of estimating the common secondary structure between two homologous RNA sequences.

It is worth noting that even though the present effort focuses entirely on Dynalign, the methodology is more general. The computed alignment constraints can be utilized with other packages for joint sequence alignment and structure prediction that permit their use for computational simplification, e.g. as "alignment envelopes" in StemLoc [11,12].

The rest of the paper is organized as follows. Section 2 provides background on the problem of RNA structure prediction and references to current related work in this area. The proposed methodology for determining alignment constraints based on nucleotide alignment posterior probabilities is summarized in Section 3. Results evaluating the performance of the proposed method and comparisons with other techniques are presented in Section 4. Section 6 summarizes the main findings of the paper. Details of our algorithmic methodology are included in Section 7.

## 2 Overview of RNA Secondary Structure Prediction Methods

RNA structure is hierarchical [13]. The *primary structure *comprises a linear chain of nucleotides joined together by covalent phosphodiester bonds. This is identified by the ordered sequence of nitrogenous bases that determine the four types of individual nucleotides: adenine (A), guanine (G), cytosine (C), and uracil (U). This primary structure is determined by "sequencing" and is the starting point of most computational methods for structure estimation. The nucleotides within a chain interact through the formation of hydrogen bonds between the pairs *A *– *U*, *G *– *C*, and *G *– *U*. The set of these base pairings is referred to as the *secondary structure*. *Tertiary structure *includes structural contacts arising from additional interactions on top of secondary structure. These define the three-dimensional structure of the RNA. Quaternary structure is the interaction with other molecules, such as with proteins or other strands of RNA. Secondary structure contacts are stronger [14-16] and form faster [17] than tertiary structure contacts, therefore secondary structure can be largely determined without knowledge of tertiary structure.

Comparative sequence analysis can be used to accurately determine the secondary structure of functional RNAs for which there are a large number of known homologs [3]. The secondary structure is the common structure to all homologs, as determined by an alignment of the structures. Over 97% of base pairs predicted for ribosomal RNA in this manner were subsequently found in high resolution crystal structures [18].

There has been a long-standing interest in the prediction of secondary structure for a single sequence and free energy minimization methods are currently the most accurate and popular. Using dynamic programming algorithms, the lowest free energy structure is determined according to a set of nearest neighbor parameters that predict conformational stability [19-23]. These parameters were empirically derived to fit stabilities determined by optical melting experiments on small model systems [19,24,25]. The accuracy of free energy minimization has been benchmarked as high as 73% for predicting known base pairs for a diverse set of sequences as long as 700 nucleotides with structures determined by comparative analysis [19], For different sequences, including longer sequences, the average accuracy is lower [26,27]. The use of dynamic programming algorithms to predict low free energy structures has been recently reviewed [28,29].

A recently introduced alternative to free energy minimization is the determination of the most probable structure using a stochastic context-free grammar (SCFG) [1]. The transition probabilities for the SCFG are trained on sets of sequences with known structure. An SCFG has been reported that is nearly as accurate as structure prediction as free energy minimization [26].

To improve the accuracy of structure prediction, a number of methods have been developed that determine a secondary structure common to two or more sequences. These methods have the advantage of including comparative data, and, in general, they fall into three classes. The first class takes a fixed sequence alignment as input. A second class predicts structures for each sequence and then sorts through the structures to find those common to all sequences. The third class finds the structure common to two or three sequences by simultaneously aligning the sequences and finding the most likely structure. In general, the third class is the most rigorous and accurate, but also the most time-consuming. A number of these methods have been reviewed [29,30].

Algorithms that find the secondary structure common to the multiple, unaligned sequences are either genetic [31] or dynamic programming algorithms. The dynamic programming approaches trace their lineage to the theoretical paper by Sankoff [4], which provides for a computational complexity of *O*(*N*^6^) in time and *O*(*N*^4^) in memory where *N *is the length of the smaller of the two sequences. A number of different implementations of the Sankoff algorithm have been developed to find the secondary structure common to two sequences. Each of these implementations restricts the search space to make the program runtime feasible. The first, FOLDALIGN [32], maximized an empirical score and originally did not allow multibranch loops to improve runtime. FOLDALIGN was later revised to allow multibranch loops and use a subset of the free energy nearest neighbor parameters [33]. The revised FOLDALIGN restricts the alignment space to reduce runtime. Two SCFG-based programs are available, StemLoc package [11,12] and Consan [34]. To reduce runtime, StemLoc introduced the concept of fold and alignment envelopes that restrict, respectively, the search space for possible base pairs and possible nucleotide alignments. In the structure domain, the set of allowed base pairs for each sequence are those found by the prediction of set of probable structures for each single sequence. For the alignment domain, the allowed nucleotide alignments are those found in the "*N *most probable" sequence alignments for some choice of *N*. In a recent similar advance [34], Consan improves its runtime by using highly probable nucleotide alignments as "pins" at which the exact alignment is constrained. Highly probable nucleotide alignments, exceeding a specified threshold, are forced to occur in the simultaneous prediction of the alignment and common sequence.

Dynalign [5,6] is an implementation of the Sankoff algorithm that predicts the lowest free energy structure common to two sequences. To make the calculation time tractable, a restriction of the alignment domain was introduced [5]. In the most recent publication [35], for nucleotide *i *from sequence one to align to *k *from sequence two, the following constraint must be met:

(1) $$|\frac{i\times N_{2}}{N_{1}}-k|\leq M$$

where *M *is a user-specified parameter, *N*_1 _is the length of sequence one, and *N*_2 _is the length of sequence two. This restriction on maximum insertion length imposes a heuristic constraint on possible alignments. This implies a constraint on the maximum insertion length, which is reasonable for homologous sequences [5]. Equation (1) corresponds to a restricted search interval in the 2^nd ^sequence for alignment of *i*^th ^nucleotide in the 1^st ^sequence. The restriction reduces the computation burden since *M *can typically be chosen much smaller than the shorter sequence length *N*. The computations are more tractable with *M *parameter heuristic: *O*(*M*^3^*N*^3^) in time and *O*(*M*^2^*N*^2^) in memory (as compared with *O*(*N*^6^) and *O*(*N*^4^) in time and memory, respectively, without the *M *parameter) where *M is *the measure of maximum permissible insertion length and *N *is length of shorter sequence. Dynalign also restricts the set of allowed base pairs for each sequence to those found in low free energy structures by single sequence secondary structure prediction [35].

In this paper, a new method for estimating alignment constraints is proposed and incorporated in Dynalign. Instead of using the most probable sequence alignments the method utilizes the *a posteriori *probabilities for nucleotide alignments in order to establish the alignment constraints. This method makes Dynalign more robust by eliminating the need for the *M *parameter above.

## 3 Alignment Constraints from Posterior Probabilities

The *M *parameter mediates a trade-off between the computation and accuracy. A smaller value of the parameter is desirable in order to reduce the computation time to practically useful values. On the other hand, a small value can be overly-restrictive and thereby reduce the accuracy of the structure and alignment prediction from Dynalign. Thus, an 'educated guess' for the *M *parameter is vital to the accuracy of secondary structure prediction. The value of *M *has hitherto been empirically determined and found to vary over different RNA families. The lack of an analytical methodology for determining the *M *parameter has been a limitation for Dynalign. In addition, a large *M *parameter is typically required for longer sequences since they typically can have longer insertions. This tends to make the computation time for Dynalign with the *M *parameter particularly onerous for longer sequences.

**Figure 1:**
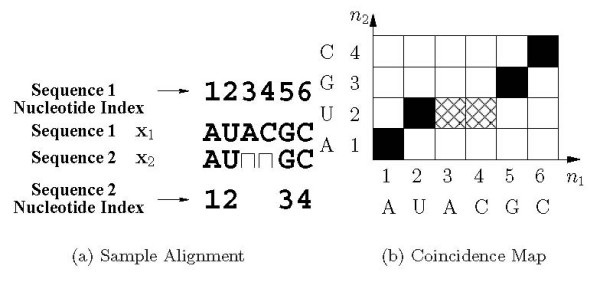
Example illustrating *co-incidence*. (a) A sample alignment of two sequences, where inserted locations in a sequence are shown with a gap ⊓ in the other sequence in the corresponding location. (b) The set of *co-incident *position pairs is depicted. Coordinates corresponding to the co-incident position pairs are indicated by black (aligned) or cross-hatched (insertion) blocks.

In this paper, a new principled methodology is proposed for the introduction of alignment constraints in Dynalign. Instead of the heuristic *M *parameter, alignment constraints are determined through a probabilistic analysis. For this purpose, the alignment between homologous sequences is modeled by a Hidden Markov Model (HMM). Using the model, the posterior probability *P*(*n*_1 _↔ *n*_2 _| **x**_1_, **x**_2_) is determined, which denotes the probability that nucleotide position *n*_1 _in the first sequence **x**_1 _is *co-incident *with nucleotide position *n*_2 _in the second sequence **x**_2_, given that the sequences are produced by the model. Two nucleotide positions (one from each of the two sequences) are said to be *co-incident *if they are either aligned, or if one nucleotide position (from one of the sequences) occurs in an insertion in that sequence that begins at a nucleotide position aligned with the second nucleotide position (from the other sequence). Formally, a nucleotide position *i *from the first sequence and nucleotide position *j *from the second sequence are said to form a co-incident pair (*i*, *j*) if: a) nucleotide positions *i *and *j *are aligned, or b) nucleotide position *i *occurs in an insertion in the first sequence and nucleotide position *j *in the second sequence aligns with nucleotide position *i*_ from the first sequence, where *i*_ denotes the largest position index less than *i *in the first sequence that aligns with a nucleotide position from the second sequence, or c) nucleotide position *j *occurs in an insertion in the second sequence and nucleotide position *i *in the first sequence aligns with nucleotide position *j*_ from the second sequence, where *j*_ denotes the largest position index less than *j *in the second sequence that aligns with a nucleotide position from the first sequence. As an example, consider the alignment shown in Figure 1(a). The co-incident nucleotide position pairs for this alignment are: (1,1), (2,2), (3,2), (4,2), (5,3), (6,4). Figure 1(b) illustrates the map of co-incident positions in the alignment of Figure 1(a) in a planar matrix of blocks, where the nucleotide position *n*_1 _for the first sequence indexes the abscissa of the block and the nucleotide position *n*_2 _indexes the ordinate of the block. In this graphical matrix representation, co-incident positions indicated above are depicted in Figure 1(b) as black blocks (for aligned locations) or cross-hatched blocks (for insertions). Figure 1(b) illustrates that for any alignment the set of co-incident positions defines an unbroken path from the lower corner of the matrix to the upper corner of the matrix (see Remark 1 in Appendix Section 8). In the dynamic programming step in Dynalign, at any given point, the sequence alignment component of the iteration searches over positions of the matrix that are adjacent to the current location (see Remark 2 in Appendix Section 8). For an alignment to be allowable, constraints specified in Dynalign (and also in alternate methods for joint structure prediction over multiple-sequences) must allow all the co-incident position pairs corresponding to the alignment.

Now, if *P*(*n*_1 _↔ *n*_2 _| **x**_1_, **x**_2_) is small, correspondingly it is rather improbable that the nucleotide position *n*_1 _in the first sequence **x**_1 _will be co-incident with nucleotide *n*_2 _in the second sequence **x**_2_. This suggests that alignments (in Dynalign) may be constrained by excluding highly improbable alignments as indicated by extremely low values of the posterior co-incidence probability. Correspondingly, an alignment constraint may be defined by thresholding the posterior co-incidence probability with a suitably low threshold *P*_thresh_. Formally, an alignment constraint set is defined as

(2) **C **= {(*n*_1_, *n*_2_) | P(*n*_1 _↔ *n*_2 _| **x**_1_, **x**_2_) > *P*_thresh_}

where **C **denotes the alignment constraint set. Elements of **C **represent nucleotide position pairs that may co-incide between the sequences and elements outside of **C **are nucleotide alignment position pairs whose co-incidences are disallowed.

**Figure 2:**
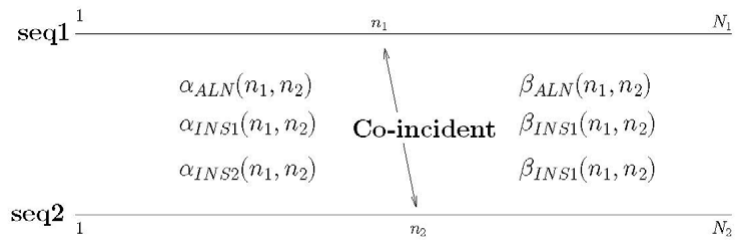
Illustration of alignment of nucleotide at *n*_1 _in 1^st ^sequence and nucleotide at *n*_2 _in 2^nd ^sequence and how forward and backward variables are related to alignment of *n*_1 _and *n*_2_. Forward variable keeps track of events before alignment position (*n*_1_, *n*_2_) and backward variable keeps track of events after alignment position (*n*_1_, *n*_2_).

The posterior probabilities of co-incidence between nucleotide positions are efficiently computed using the HMM forward-backward algorithm. The process is schematically illustrated in Figure 2. The posterior probability is computed in terms of forward and backward variables as

(3) $$P(n_{1}↔n_{2}|x_{1},x_{2})=\frac{\sum_{m}\alpha_{m}(n_{1},n_{2})\beta_{m}(n_{1},n_{2})}{P(x_{1},x_{2})}$$

where the sum is over the three possible states for nucleotide co-incidence, i.e., *m *= *ALN*, *INS*1, *INS*2, the forward variable *α*_*m*_(*n*_1_, *n*_2_) represents the probability that the subsequences x11n1 and x21n2 of the first and second sequence, respectively, are produced and the nucleotide positions *n*_1 _and *n*_2 _are in the state *m*, and the backward variable *β*_*m*_(*n*_1_, *n*_2_) represents the probability that subsequences x1n1+1N1 and x2n2+1N2 are observed given that the $n_{1}^{th}$ and $n_{2}^{th}$ nucleotide positions are in state *m*. Details of the model and the computation of posterior probabilities are provided in Section 7.

**Figure 3:**
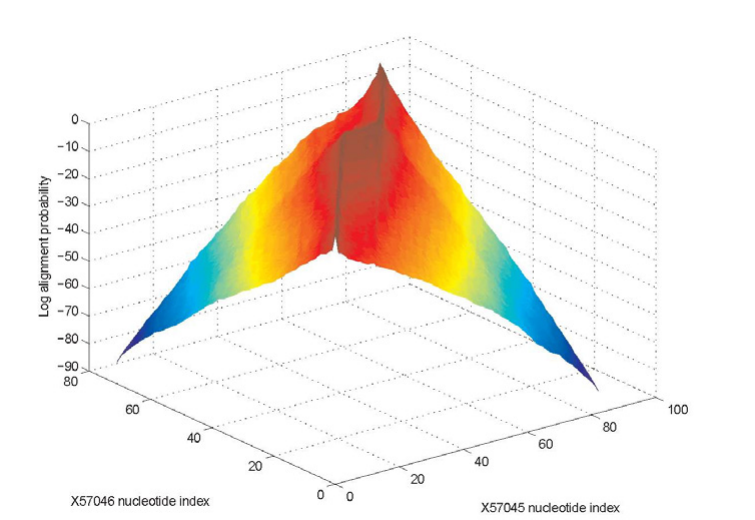
Logarithm of posterior probabilities for co-incidences of nucleotide positions for a pair of sequences in a surface plot representation.

Figure 3 shows a surface plot of the (logarithm of) the posterior probability in (3) as a function of sequence nucleotide indices *n*_1 _and *n*_2_, for an exemplary case of two homologous RNA sequences. At locations where the "probability surface" is close to unity, there is good confidence that the corresponding nucleotide positions are co-incident and conversely near zero values of the "probability surface" reflect near certainty that the corresponding nucleotide positions are not co-incident as per the model. The process of determining the alignment constraint set in (2), may be thought of as slicing the "probability surface" Figure 3 at a sufficiently low level close to the (*n*_1_, *n*_2_) plane.

The choice of the threshold *P*_thresh _in Eqn (2) represents a trade-off between computation and the accuracy of the alignment constraint (in a manner analogous to the *M*-parameter). A smaller *P*_thresh _offers higher confidence that all actual alignments will lie within the corresponding constraint sets but also requires more computation due to the increased choices and region of computation in the Dynalign phase. A high value of this threshold, on the other hand, results in more a stringent alignment constraint set and thereby reduced computation requirements. However, there is a higher probability that actual alignments will not lie within the constraint set, whereby the prediction accuracy for Dynalign is (likely to be) reduced (see Remark 3 in Appendix Section 8).

Suitable HMM parameter and threshold values are required in order to determine effective constraint sets. In order to have parameters that accurately capture statistical characteristics, sequence pairs are grouped into bins based on the percentage of nucleotides that are identical in the optimal (maximum-likelihood) alignment and the parameter and threshold values are established for each bin. This ensures that the diverse range of statistical variation observed in sequence pairs is divided up into clusters where the parameters for each cluster better represent the statistics of sequence pairs in the cluster than would be feasible with a single global model. This process is analogous to binning employed in previously published work [11,36], Details of the binning and parameter estimation can be found in Section 7.

**Figure 4:**
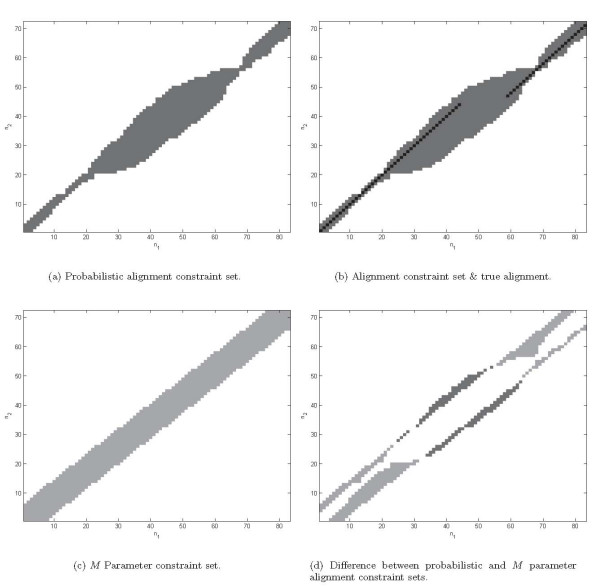
Illustration of the probabilistic alignment constraint set and comparison against the true alignment and the *M *parameter constraint set for tRNA sequences: X57045 and X57046. The abscissa and ordinates of the plots indicate nucleotide positions *n*_1 _and *n*_2 _along the sequences X57045 and X57046, respectively, (a) Probabilistic alignment constraint set with permitted alignments (shown in dark gray). (b) Alignment constraint set with true alignment super-imposed (in black). (c) Alignment constraint set for the prior *M *parameter with *M *= 7 (shown in light gray). (d) Difference between the *M *parameter and the probabilistic alignment constraint sets. Light gray regions indicate nucleotide position alignments permitted by the *M *constraint and not by the probabilistic constraint and the situation is vice-versa for dark gray regions.

A sample alignment constraint set obtained for two tRNA sequences, X57045 and X57046, is shown in Figure 4, where the constraint set is also compared against the actual (hand curated) alignment from RFAM database [37] and the constraints implied by Eqn. (1) for a value of *M *= 7. In Figure 4(b), the true alignment in black clearly indicates an insertion run in the second sequence. It is also clear that the probabilistic alignment constraint set includes the true alignment (as desired). A comparison of Figs. 4(a) and 4(c) is also instructive: while the *M *parameter constraint allows a uniform band of nucleotide alignments about the "diagonal" line, the probabilistic alignment constraints are adaptive to the confidence in the alignment and provide tighter constraint in regions where this confidence is high and a looser constraint where this confidence is low (in the vicinity of the insertion run). Finally, Figure 4(d), illustrates the difference between the probabilistic and the *M*-parameter constraint sets. The significantly larger light gray area in comparison to the dark gray area in this figure illustrate the computational savings of this method in comparison to the *M*-parameter constraint.

## 4 Results

Three sets of experiments are performed: 1) Experiments for verifying accuracy of probabilistic alignment constraints, 2) Experiments for determining structural prediction accuracy, and 3) Experiments for comparing the computation and memory requirements. The latter two sets of experiments also compare performance of Dynalign with the new constraint proposed here against the previous version of the Dynalign (with the *M *parameter constraint) and against other secondary structure prediction methods. The parameters for the algorithms and software version numbers used are summarized in Section 7.6.

### 4.1 Accuracy of Probabilistic Alignment Constraints

The experiments for accuracy of probabilistic alignment constraints are performed over 5S RNA and tRNA alignments in the RFAM database [37]. The accuracy is determined by how probabilistic alignment constraint performs with respect to actual annotated alignment in RFAM database. Sensitivity and specificity of alignment constraint will be used to report accuracy of probabilistic alignment constraint and compare it with the *M *parameter alignment constraint. Sensitivity for alignment constraint accuracy is defined as:

(4) $$\text{sensitivity}=\frac{N_{tp}}{N_{tp}+N_{fn}}$$

where *N*_*tp *_is number of true positive predictions corresponding to number of alignment positions which are in actual (annotated as in RFAM database) alignment and within the alignment constraint set. *N*_*fn *_is number of false negative predictions corresponding to number of alignment positions which are outside alignment constraint set but are in the annotated alignment. Sensitivity is therefore the fraction of known alignment positions that are allowed in the alignment constraint set.

Specificity for alignment constraint accuracy is similarly defined as:

(5) $$\text{specificity}=\frac{N_{tn}}{N_{tn}+N_{fp}}$$

where *N*_*tn *_is number of true negative predictions (i.e. not in RFAM annotation) corresponding to alignment positions which are outside the probabilistic alignment constraints. *N*_*fp*_is number of false positive predictions corresponding to alignment positions which are alignment constraint but are not annotated as aligned in RFAM. Specificity, therefore, is the fraction of alignments known to not occur that are not allowed in the alignment constraint set.

Since the alignment constraint serves as a "pre-filter" in Dynalign, a high sensitivity is desirable even at the cost of some degradation in specificity (see Remark 4 in Appendix Section 8). In order to better distinguish between high specificity values, the fraction of nucleotide alignment positions missed are computed and tabulated in Table 1 far the proposed probabilistic alignment constraints and the *M *parameter constraints, used with *M *= 7 [35]. The values in Table 1 indicate that the proposed method for determining alignment constraints offers very high sensitivity and performs significantly better than the *M *constraint that was previously employed. At the same time, the proposed method also has better average specificity than the *M *parameter. Note also that the hand curated alignments in the RFAM database include some nucleotide position alignments that are obtained using considerations that are exogenous to sequence nucleotide similarity, e.g. by structural alignments. This, in part, limits the sensitivity that may be obtained using most methods based on sequence nucleotide similarity alone (including ours).

**Table 1:** Accuracy of alignment constraints: Fraction of total number of alignment positions missed in 619941 tRNA pairwise alignments and 180901 5S RNA pairwise alignment in RFAM database.

		New constraint	*M *constraint
Fraction of alignment positions missed	tRNA	2.2 × 10^-4^	2.9 × 10^-3^
	5S RNA	2.0 × 10^-4^	4.4 × 10^-3^

For the *M *constraint a value of *M *= 7 is chosen is chosen as established in [35]. New constraint refers to the probabilistic alignment constraint proposed in this paper.

Table 2 summarizes the average values of sensitivity and specificity over tRNA and 5S RNA alignments in RFAM database for the proposed probabilistic alignment constraint, the previously employed *M *constraint, and for single sequence prediction [19]. Once again, the average values indicate the superior performance of the proposed method over the previous *M *constraint. The maximum and minimum values indicate the limits of the performance of these two methods. Note that the probabilistic alignment constraint exhibits greater variability in the minimum and maximum specificity as can be expected due to the adaptive nature of the constraint.

**Table 2:** Average, minimum, maximum sensitivity and specificity over tRNA and 5S RNA alignments in RFAM database for the proposed probabilistic alignment constraint (New constraint) and the previously employed *M *constraint.

		New constraint		*M *constraint	
		
		sensitivity	specificity	sensitivity	specificity
**tRNA**	avg	0.999	0.827	0.997	0.816
	min	0.38	0.42	0.44	0.78
	max	1.00	0.99	1.00	0.85
**5S RNA**	avg	0.999	0.924	0.995	0.883
	min	0.78	0.63	0.53	0.85
	max	1.00	0.98	1.00	0.88

### 4.2 Accuracy of Structural Prediction

An evaluation of the structural prediction accuracy obtained with the proposed constraints is important in order to establish that the constraints are not overly stringent. For this purpose, an archive containing 309 5S RNAs [38] and 484 tRNAs [39] with known secondary structures is utilized. Three methods for secondary structure prediction are compared over 2000 randomly selected 5S RNA and 2000 randomly selected tRNA sequence pairs from this archive: a) Dynalign with probabilistic alignment constraints, b) Dynalign with *M *constraint with *M *= 7 and c) single sequence structure prediction [19].

Structural prediction accuracy for each of the methods is quantified in terms of sensitivity and positive predictive value (PPV). Sensitivity is defined as the fraction of canonical pairings in the known (or true) structure that are correctly predicted and PPV is defined as the fraction of predicted base pairings that are in agreement with the known structure. In both cases, a single nucleotide shift on any one side is allowed. Detailed definitions of the sensitivity and PPV can be found in the methods part in Section 7.5.

The average values of PPV and sensitivity for the three methods are listed in Tables 3 and 4 for the tRNAs and 5S RNAs, respectively. The tables for the tRNA dataset include two columns indicated by + and - that correspond, respectively, to versions of the algorithms that do or do not utilize available knowledge of modified nucleotides that cannot accommodate the canonical A-form helix. For the results in the + columns, these nucleotides are forced single-stranded to improve structure prediction [24] whereas the information is not utilized for the results in the – column. From the tabulated numbers it is clear that Dynalign with the proposed probabilistic alignment constraint is improved compared to the previously used *M *constraints. Both Dynalign methods outperform single structure prediction.

**Table 3:** Structural prediction accuracy of Dynalign with the *M *constraint and the new constraint, and for single sequence prediction over 2000 random tRNA pairs.

	Dynalign					
			
	New constraint		*M *constraint		Single Sequence Prediction	
	
	+	-	+	-	+	-
Sensitivity	0.926	0.873	0.923	0.866	0.874	0.764
PPV	0.925	0.846	0.922	0.836	0.848	0.707

Column with '+' refers to Dynalign making use of structural constraints where modified nucleotides that cannot accommodate the canonical A-form helix are forced single-stranded [24].

**Table 4:** Structural prediction accuracy of Dynalign with *M *constraint, new constraint and single prediction over 2000 random 5S RNA pairs.

	Dynalign		
		
	New constraint	*M *constraint	Single Sequence Prediction
Sensitivity	0.907	0.905	0.739
PPV	0.821	0.817	0.647

### 4.3 Computation and Memory Requirements

In order to compare the computational and memory requirements for Dynalign with the proposed probabilistic alignment constraints against the prior *M*-parameter heuristic, these requirements were estimated by sampling 100 tRNA and 5S RNA sequence pairs each at random from the RFAM database and recording the CPU time and memory usage. An Opteron 270 (dual core, 2 GHz) machine with 4 GB of RAM using Fedora Core running Linux Core 4 and gcc compiler were utilized for these experiments. For the *M *parameter constraint a value of *M *= 7 was used throughout as established in the most recent publication on Dynalign [35].

Minimum, maximum, and average CPU times per Dynalign execution (for a sequence pair) are reported in Table 5. The CPU time in these tables is as reported by *Linux *and it refers to time in seconds that the (Dynalign) process occupies the CPU excluding dispatches and input/output wait times. The major benefit of the proposed methodology is immediately apparent from the numerical figures in this table. On average, the incorporation of the probabilistic constraints reduces the CPU time by a factor of approximately 2 for the 5S RNA experiments, where the average sequence length was 119.59 nucleotides. For the tRNA experiments, where the average sequence length was much shorter (77.44 nucleotides), the method requires almost the same computation time as the previous version of Dynalign (with the *M *parameter). In fact, the computational time requirement for this case favors the *M*-parameter Dynalign by a small amount. This is the result of computational optimizations that have been incorporated in Dynalign due to which the computation is now significantly faster than prior versions. As a result of this speed-up, for smaller sequences, the overhead of computing the alignment envelope for the new method and of performing the resulting dynamic memory allocations is no longer negligible in comparison to the overall run time, whereas in the *M *constraint, these are pre-determined.

**Table 5:** Average, minimum, maximum run times (in seconds) and memory (in megabytes) requirement results of proposed probabilistic alignment constraint (New constraint) and the previously employed *M *constraint.

		tRNA		5S RNA	
		
		New constraint	*M *constraint	New constraint	*M *constraint
**Memory**	avg	10.988	10.960	12.377	14.306
	min	9.36	10.528	10.176	12.664
	max	13.592	12.040	17.436	14.840
**Timing**	avg	9.98	9.39	34.38	73.07
	min	1.0	4.0	20.0	36.0
	max	55.0	29.0	234.0	111.0

A dual-core AMD Opteron^®^-270 2.0 GHz system with 4 GBytes of main memory running Linux Fedora Core 4 was utilized for the timing experiments.

The significance of these timing gains is even greater when taken in conjunction with the results in the preceding sections that indicate that the improvement is not at the cost of prediction accuracy. Furthermore, it is worth noting that the speed-up factor is larger for the longer 5S RNA sequences. Since these require significantly more time than the shorter tRNA sequences the overall impact of the speed-up is very significant and in fact increases the length of sequences on which Dynalign can be deployed.

The memory requirements for the two methods are compared in Table 5, where the minimum, maximum, and average memory (in megabytes) required for the 100 sequence pairs each of tRNAs and 5S RNAs are indicated. Memory requirements are as reported in the *size *entry of *Linux ps *command after all requisite dynamic allocations are done. This number corresponds to approximate value of virtual memory usage of the (Dynalign) process. The tabulated numbers indicate that the proposed method also offers a memory advantage. As might be anticipated, the advantage is relatively minor for short sequence lengths (e.g. tRNAs) but quite significant for longer sequences (e.g. 5S RNAs). The savings for longer sequences are particularly attractive since often memory is a limitation that restricts the length of sequences for which Dynalign may be utilized.

### 4.4 Benchmarking Against Other Structure Prediction Methods

For benchmarking purposes, the implementation of the new principled alignment constraint (using the threshold of posterior co-incidence probabilities) and the previous Dynalign banding constraint (*M *constraint) [35] were also compared against three other dynamic programming algorithms. The three other programs are FOLDALIGN [33], which uses a free energy-based scoring function, and StemLoc [11] and Consan [34], which use SCFG models. Each program was run using default parameters. Our interest is both in the accuracy of the methods and in their use of resources, CPU time and memory.

Tables 6 and 7 show the accuracy of structure prediction for the methods with tRNA [39] and 5S rRNA [38], respectively. In each case, 2000 pairs of sequences were randomly chosen from a database of sequences with known structure. These tables also show the comparison of the programs that find the structure common to two sequences to the accuracy of single sequence secondary structure prediction. Sensitivity and positive predictive value (PPV) are both scored [22], where sensitivity is the fraction of known pairs correctly predicted and positive predictive value is the fraction of predicted pairs in the known structure. Results are stratified by percent similarity of the two sequences and the final column summarizes the results over all values of sequence pair similarities.

**Table 6:** Structural prediction accuracy statistics for the methods benchmarked over 2000 random tRNA selections.

		Percent sequence similarity				
		
		20–40	40–60	60–80	80–100	0–100
**Dynalign new constraint**	Sens	0.830	0.874	0.936	0.719	0.873
	PPV	0.800	0.845	0.930	0.701	0.846
**Dynalign *M *constraint**	Sens	0.824	0.867	0.930	0.719	0.866
	PPV	0.792	0.834	0.921	0.701	0.836
**FOLDALIGN**	Sens	0.753	0.858	0.898	0.690	0.848
	PPV	0.838	0.906	0.949	0.846	0.902
**StemLoc**	Sens	0.587	0.896	0.943	0.871	0.862
	PPV	0.755	0.901	0.926	0.876	0.889
**Consan**	Sens	0.800	0.909	0.945	0.768	0.899
	PPV	0.769	0.852	0.873	0.667	0.843
**Single Prediction**	Sens	0.757	0.762	0.785	0.717	0.764
	PPV	0.709	0.703	0.738	0.673	0.707

Results are summarized for sequence similarity values ranging from 20% through 100% in steps of 20% and for the overall data set (0 – 100). Dynalign new constraint refers to Dynalign with probabilistic alignment constraints proposed here. Software version numbers and parameters for the algorithms are described in Section 7.6.

**Table 7:** Structural prediction accuracy statistics for the methods benchmarked over 2000 random 5S RNA selections.

		Percent sequence similarity				
		
		20–40	40–60	60–80	80–100	0–100
**Dynalign new constraint**	Sens	0.895	0.903	0.914	0.909	0.907
	PPV	0.838	0.824	0.821	0.785	0.821
**Dynalign *M *constraint**	Sens	0.892	0.901	0.912	0.909	0.905
	PPV	0.837	0.821	0.815	0.786	0.817
**FOLDALIGN**	Sens	0.726	0.753	0.787	0.510	0.749
	PPV	0.772	0.784	0.803	0.572	0.778
**StemLoc**	Sens	0.272	0.673	0.899	0.776	0.740
	PPV	0.652	0.805	0.901	0.826	0.840
**Consan**	Sens	0.666	0.799	0.931	0.865	0.842
	PPV	0.671	0.761	0.817	0.722	0.775
**Single Prediction**	Sens	0.680	0.722	0.766	0.784	0.739
	PPV	0.603	0.632	0.672	0.673	0.647

Results are summarized for sequence similarity values ranging from 20% through 100% in steps of 20% and for the overall data set (0 – 100). Dynalign new constraint refers to Dynalign with probabilistic alignment constraints proposed here. Software version numbers and parameters for the algorithms are described in Section 7.6.

For determining computation and memory resource requirements, calculations were performed using one core on a dual Opteron 270 (dual core, 2 GHz) machine with 4 GB of RAM under Fedora Core Linux with the gcc compiler.

Table 8 compares the computation times for the different methods benchmarked in this study and the memory requirements for these methods are listed in Table 9. Dynalign performs favorably as compared to the other programs. Each method has similar average accuracy on the tRNA database. The free energy minimization approaches, Dynalign, FOLDALIGN and single sequence prediction, provide lower accuracy than the SCFG-based methods at high sequence identity and higher accuracy at low sequence identity. For 5S rRNA, however, Dynalign has higher average accuracy than all the other methods tested. In particular, Dynalign does well on cases with both high and low pairwise identity. In general, the SCFG-based methods show lower average accuracy as pairwise identity decreases. Each structure prediction method that finds the structure common to two sequences is significantly more accurate than single sequence secondary structure prediction. Dynalign is faster and uses less memory than the other programs.

**Table 8:** Minimum, Maximum and Average run times (in seconds) for 5 different methods over 100 randomly chosen 5S RNA and tRNA alignments each from [38] and [39].

	tRNA			5S RNA		
	
	Min	Max	Avg	Min	Max	Avg
**Dynalign new constraint**	1.0	55.0	9.98	2.0	234.0	34.38
**Dynalign *M *constraint**	4.0	29.0	9.39	36.0	111.0	73.07
**StemLoc**	3.0	1308.0	210.16	11.0	8133.0	616.06
**Consan**	21.0	793.0	209.29	123.0	7330.0	1032.84
**FOLDALIGN**	14.0	76.0	30.33	181.0	822.0	349.48

A dual-core AMD Opteron^®^-270 2.0 GHz system with 4 GBytes of main memory running Linux Fedora Core 4 was utilized for the timing experiments.

**Table 9:** Memory requirements (in megabytes of main memory) 5 different structure prediction methods on 5S RNAs and tRNAs alignments from [38] and [39]

	tRNA			5S RNA		
	
	Min	Max	Avg	Min	Max	Avg
**Dynalign new constraint**	9.360	13.592	10.988	10.176	17.436	12.277
**Dynalign *M *constraint**	10.528	12.040	10.960	12.664	14.840	14.306
**StemLoc**	41.440	750.660	252.246	94.300	2788.296	415.973
**Consan**	34.272	358.704	131.595	98.976	1676.492	317.303
**FOLDALIGN**	93.276	230.032	134.438	585.900	820.204	730.214

A dual-core AMD Opteron^®^-270 2.0 GHz system with 4 GBytes of main memory running Linux Fedora Core 4 was utilized for the timing experiments.

## 5 Discussion

In this paper, a novel technique is presented for reducing the alignment search space for finding the common secondary structure and alignment for two RNA sequences. The allowed nucleotide alignments are those determined to be reasonable (posterior nucleotide co-incidence probability greater than a chosen threshold) as determined by a forward-backward calculation using a Hidden Markov Model (HMM). This new method provides a significant improvement in rigor and speed as compared to previous versions of Dynalign, in which the user was required to empirically choose a parameter that set a band of allowed alignments [5,35]. The HMM is significantly more flexible in the application of constraints because the allowed region is narrow where the alignment is well-defined by sequence conservation alone, but also wide when the alignment is poorly defined.

Other pairwise implementations of the Sankoff algorithm have also explored alignment constraints to speed execution time. For StemLoc, Holmes introduced the alignment "envelope," which is the set of allowed nucleotide alignments as determined by the union of "N-best alignments," i.e. only nucleotide position alignments occurring in the *N *most likelihood sequence alignments [11,12]. This has the disadvantage that regions of the nucleotide alignment can be poorly resolved and therefore not well sampled with *N *alignments. For Consan, Dowell and Eddy use a forward-backward HMM to determine nucleotide alignments of high probability, called "pins" [34]. In Consan, these high probability nucleotide alignments are forced to occur. This approach has the drawback that there are no constraints between pins and, for sequence pairs with low identity, it is possible that no pins will be found.

The present manuscript describes the combination of the proposed method for determining alignment constraints with Dynalign. The technique, however, is general and would apply to other implementations of the Sankoff algorithm, including Consan [34], FOLDALIGN [33], PMcomp [40], and StemLoc [11]. Two pairwise calculations of alignment constraints could also be used to accelerate X-Dynalign, a program that finds the common structure and alignment for three homologous sequences [41]. Aside from the improved speed of Dynalign for determining secondary structure, this new method will have a significant impact in the discovery of non-coding RNA (ncRNA) sequences in genome scans. Two papers have reported the use of algorithms that can find the secondary structure common to two unaligned sequences as generic RNA gene finders [35,42]. These programs have high sensitivity for ncRNAs, especially for regions of genome alignment that have low pairwise identity. The principal drawback is that they are slow compared to programs that find ncRNAs by scanning fixed, predetermined alignments [43,44]. The method used here to accelerate Dynalign mitigates some of the computational cost and will allow faster scanning of genomes for novel ncRNAs. The success of the methodology also suggests that iterative methods based on *a posteriori *probability estimates that have been extremely successful in communication systems [8,9] may offer parallels in biological problems involving sequence and structure similarity [45].

Since the method adaptively computes alignment constraints for each pair of input sequences, its complexity varies depending on the input sequence pair. In practice, the computational complexity will depend on the extent of sequence conservation between the two homologous sequences. In the worst case, it can require as much computation as the full unrestricted Sankoff algorithm, viz. *O*(*N*^6^) in computation and *O*(*N*^4^) in memory. In the best case, where the alignment constraint actually corresponds to a single alignment, i.e. a band of width *M *= 1, the complexity reduces to *O*(*N*^3^) in computation and *O*(*N*^2^) in memory. From the computational perspective, the best case scenario is encountered for the case of identical sequences with sufficient nucleotide diversity (within a sequence). In this case, however, comparative analysis offers little benefit for the overall problem of joint structure prediction. The worst case scenario is rare for homologous sequences.

Finally, note that the alignment constraint constitutes only one element (out of at least three) that determine the structural prediction accuracy. The accuracy depends also on the thermodynamic scoring model and on the "fold constraints" utilized in the computation. In particular, experimental results for some of the situations in which the proposed method fails demonstrate that the thermodynamic model actually predicts a lower free energy for the structure determined by Dynalign (with the new constraint) than for the true structure.

## 6 Conclusion

A new procedure is proposed for establishing alignment constraints in joint alignment and secondary structure prediction problems for RNA sequences. The proposed technique when integrated in Dynalign eliminates the need for manual parameter selection and provides significant computational savings (a factor of 2 over a 5S RNA database with average sequence length of approximately 120 nucleotides) while simultaneously providing a small improvement in the structural prediction accuracy. The revised version of Dynalign can be downloaded, either as source code or as part of the RNAstructure package for Microsoft Windows [46].

## 7 Methods

A Hidden Markov Model (HMM) formulation is utilized in order to estimate a *posteriori *symbol-to-symbol alignment probabilities. HMMs have been previously used in sequence analysis [1], in speech recognition [47], and in error correction coding [7].

### 7.0.1 Notation

Individual output sequences are denoted by lowercase boldface letters specifically **x**_1 _and **x**_2 _for the case of two sequences. Specific nucleotides or subsequences selected from a sequence are indicated by prescripts: xn1 denotes the *n*_1_^th ^nucleotide of the 1^st ^sequence and xn1n2 denotes the subsequence of nucleotides from index *n*_1 _to *n*_2 _in sequence **x**. An alignment between the two sequences is specified by a sequence of states from the set

(6) *M *= {ALN, INS1, INS2}

**Figure 5:**
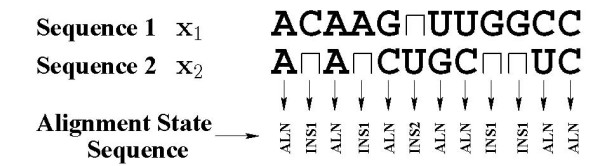
A sample sequence alignment and corresponding state sequence.

These states define an alignment by relating nucleotide positions between the two sequences as illustrated by means of an example in Figure 5. The ALN state represents *aligned *nucleotide positions, where each of the two sequences has a nucleotide (identical nucleotides in these positions are evidence of evolutionary conservation and differences represent mutations). An INS1 state represents an insertion of the first sequence, where there is a nucleotide in the first sequence **x**_1 _but no corresponding nucleotide in the second sequence **x**_2_. In Figure 5 this is denoted by pairing the nucleotide in sequence **x**_1 _with the null or *gap *symbol ⊓. Similarly, the INS2 state represents the complementary situation of an insertion in the second sequence. The letter *m *(possibly subscripted) will denote a specific state, i.e., an element of the set *M*.

A nucleotide position *n*_1 _from the first sequence is said to be *co-incident *with a nucleotide position *n*_2 _from the second sequence if either of the following three conditions hold:

1. *n*_1 _and *n*_2 _are aligned.

2. *n*_1 _occurs in an "insertion run" in the first sequence that began at a nucleotide position aligned with position *n*_2 _in the second sequence. Formally, this may be described as follows: the nucleotide at position *n*_1 _is an insertion in the first sequence and nucleotide position ${n^{\prime }}_{1}$ from sequence 1 is aligned with nucleotide position *n*_2 _from sequence 2, where ${n^{\prime }}_{1}$ denotes the largest nucleotide position index in sequence 1 under *n*_1 _that is aligned (with a nucleotide position from sequence 2).

3. *n*_2 _occurs in an "insertion run" in the second sequence that began at a nucleotide position aligned with position *n*_1 _in the first sequence, i.e., the nucleotide at position *n*_2 _is an insertion in the first sequence and nucleotide position ${n^{\prime }}_{2}$ from sequence 2 is aligned with nucleotide position *n*_1 _from sequence 1, where ${n^{\prime }}_{2}$ denotes the largest nucleotide position index in sequence 2 under *n*_2 _that is aligned (with a nucleotide position from sequence 1).

#### 7.1 Hidden Markov Model for Homologous Sequences

The HMM models the relation between two homologous nucleotide sequences, **x**_1 _and **x**_2 _by a two-stage probabilistic model. The first stage comprises the sequence of states that represents the alignment and the second stage models the nucleotides in the sequences. Specifically, the sequence of states in the first stage of the model are assumed to constitute a time-invariant (see Remark 5 in Appendix Section 8).

**Figure 6:**
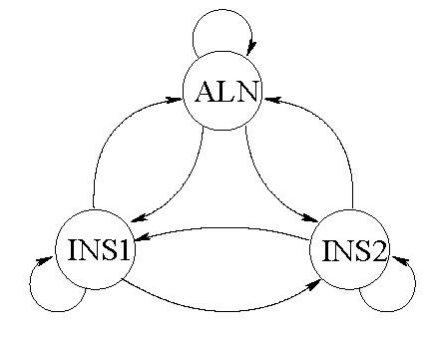
State transition diagram for the (hidden) Markov Process determining alignment between the sequences. The three states ALN, INS1, and INS2 represent alignment, insertion in sequence 1 and insertion in sequence 2, respectively.

Markov process. This three-state Markov process can be represented in terms of the state transition diagram illustrated in Figure 6 and is characterized by the state transition probabilities ${\left\{\tau (m_{1},m_{2})\right\}}_{m_{1},m_{2}\in M}$ where *τ*(*m*_1_, *m*_2_) ≡ *P*(*m*_2 _*| m*_1_)represents the probability that the next state is *m*_2 _given that the current state is *m*_1_. In each stage, the HMM emits an ordered symbol pair from the alphabet set {A, C, G, U, ⊓}. The chronological progression of the first elements of the emitted ordered pair constitute the first sequence and the second elements make up the second sequence. As indicated before, the ⊓ represents a null symbol or a gap where no nucleotide is emitted in the sequence. The emission probabilities for all possible ordered pairs, for each of the states, characterize the second stage of the model. The probability that the symbol pair (*u*, *v*) is emitted in state *m *is denoted by *γ*_*m*_(*u*, *v*). Each state of the alignment Markov process allows emission of only a subset of the total set of ordered symbol pairs. Ordered symbol pairs emitted in the aligned states can only be nucleotide pairs (no gaps), those emitted in the INS1 state can only be of the type (*X*, ⊓) where *X *is a nucleotide, and those emitted in the INS2 state can only be of the type (⊓, *X*) where *X *is a nucleotide. A gap pair (⊓, ⊓) is a disallowed output in any state. Disallowed outputs are readily handled in the model by requiring that the corresponding emission probabilities are zero. Observe that the ALN state can output ordered pairs which are mismatched (i.e. pairs of differing nucleotides). As a final remark on the model, note that the alignment states are in fact hidden and unobservable since only the sequences are observed.

The HMM parameters consist of the state transition probabilities for the first stage Markov process and the emission probabilities for the second stage. The model is useful because with appropriately chosen parameters it captures observed statistics of homologous sequences. For instance, in homologous sequences, aligned base pairs are more likely to have matching rather than differing nucleotides. Higher probabilities for emission of these matching nucleotide pairs in the ALN state allow for this statistical behavior to be modeled. Similarly, gaps in homologous sequences tend to occur in long runs rather than as single nucleotide insertions; a characteristic that the HMM can represent through a high probability of self transitions for the states in Figure 6. Thus the HMM provides a suitable model for homologous sequences.

The HMM formulation is advantageous because it can provide answers to several meaningful questions in a computationally tractable and efficient manner. For instance, given any two sequences one can determine the most probable alignment between them (as per the model) and the corresponding probability that sequences are related by the model. If this probability is high (and the model is good), one may infer that the sequences are likely to be homologs. This method is in fact utilized for sequence alignment and computing probabilistic similarity scores. However, as indicated in Section 1 for ncRNA sequence similarity alone is a poor metric for homology, particularly in regions with poor sequence similarity (since these may still have high structural similarity). Therefore, instead of determining the best alignment and the corresponding probability, the proposed method computes the posterior probability of each nucleotide position in one sequence being aligned with each nucleotide position in the other sequence. Subsequently, we can use these posterior probability estimates in order to restrict the search space for Dynalign and improve its computational efficiency. Specifically, as described in Section 3, an alignment constraint is computed by thresholding posterior alignment probabilities of nucleotide positions of two sequences. The estimation of these posterior alignment probabilities using the HMM is described next.

#### 7.2 Posterior Probabilities for Pairwise Sequence Alignment

The posterior probability *P*(*n*_1 _↔ *n*_2_*| ***x**_1_, **x**_2_) in (3) denotes the probability that nucleotide position *n*_1 _in the first sequence **x**_1 _is co-incident with nucleotide position *n*_2 _in the second sequence **x**_2_, given that the HMM emits the two sequences. Conceptually, this probability may be determined by computing the probability of each possible alignment between the two sequences and summing up the probabilities for the alignments for which the $n_{1}^{th}$ position of **x**_1 _is co-incident with the $n_{2}^{th}$ position of **x**_2 _to obtain the desired posterior probability (and dividing by the probability that the sequences are emitted by the model). The process, however, would require computation and memory that are exponential in the sequence lengths and is infeasible for practical sequence lengths of interest. Using the HMM forward-backward algorithm, which constitutes a dynamic programming approach for the problem, these probabilities may be computed in a computationally efficient manner. The forward-backward algorithm provides a set of efficient recursions using which the posterior probabilities can be determined. For the general HMM setting, these recursions may be found in [47]. The specific situation of pairwise alignment HMMs will be considered here.

### 7.2.1 Trellis Representation of the Hidden Markov Process

**Figure 7:**
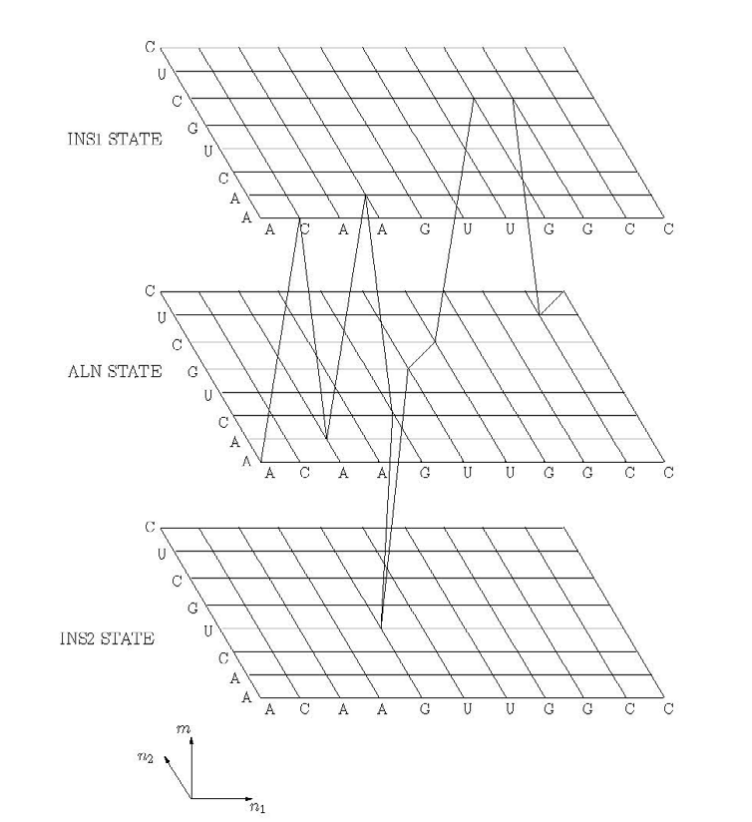
Trellis illustrating an alignment path.

In order to present the forward-backward recursions, it is convenient to represent the HMM in the form of a three-dimensional trellis of nodes {(*n*_1_, *n*_2_, *m*)}, where the first two coordinates correspond to the sequence indices and the third corresponds to the hidden state variable. An alignment between two sequences can be represented in the form of a *feasible *path in the trellis (described subsequently in this section). A sample trellis is shown in Figure 7, which depicts the nucleotide sequences and alignment path corresponding to the example of Figure 5.

The transitions between the trellis nodes represent the (hidden) Markov process for the alignment. The process can be thought of as evolving in "time" by transitioning from a *current *node position (${n^{\prime }}_{1}$, ${n^{\prime }}_{2}$, *m'*) in the trellis to the *next *node (*n*_1_, *n*_2_, *m*) and emitting a pair of symbols (for the two sequences). In this process (see Remark 6 in Appendix Section 8), the transition probabilities (for the next state) are determined by the current state and the emission probabilities (for the emitted symbol pair) are determined by the next state. Furthermore, the next node (*n*_1_, *n*_2_, *m*) is determined by the current node (${n^{\prime }}_{1}$, ${n^{\prime }}_{2}$, *m'*) and the next state *m as *follows:

(7) $$n_{1}=\{\begin{array}{ll} {n^{\prime }}_{1} & \text{if }m=\text{INS}2\\ {n^{\prime }}_{1}+1 & \text{otherwise} \end{array}$$

and

(8) $$n_{2}=\{\begin{array}{ll} {n^{\prime }}_{2} & \text{if }m=\text{INS}1\\ {n^{\prime }}_{2}+1 & \text{otherwise} \end{array}.$$

**Figure 8:**
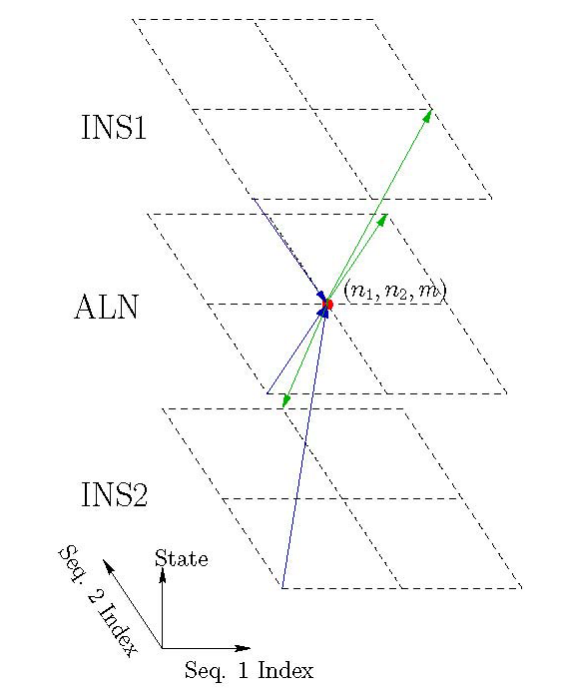
Segment of the HMM trellis illustrating the feasible edges arriving at and originating from one trellis node. The red solid circle represents the trellis node for which edges are depicted (it corresponds to an ALN state). Feasible edges arriving at the node are shown as blue arrows that converge at the trellis node and feasible edges originating from the node are shown as green arrows diverging outward from the trellis node.

These constraints reflect the state dependent constraints on emitted symbol pairs that were outlined earlier and restrict the allowable paths for the process within the trellis. Only (directed) edges (${n^{\prime }}_{1}$, ${n^{\prime }}_{2}$, *m'*) → (*n*_1_, *n*_2_, *m*) between adjacent (in a 3-D neighborhood) nodes in the trellis that satisfy the above constraint are allowed or *feasible *transitions between trellis nodes. The allowed or *feasible *paths are then a sequence of feasible edges such that the end node of each edge is identical to the beginning node of the next edge. A section of the trellis illustrating the feasible paths corresponding to a specific node is shown in Figure 8.

### 7.2.2 Forward-Backward Algorithm for Posterior Probability Computation

From Bayes' rule, the posterior probability in (3) may be written as

(9) $$P(n_{1}↔n_{2}|x_{1},x_{2})=\frac{P(n_{1}↔n_{2},x_{1},x_{2})}{P(x_{1},x_{2})}$$

where *P*(*n*_1 _↔ *n*_2_, **x**_1_, **x**_2_) represents the joint probability that nucleotide *n*_1 _of sequence 1 is co-incident with nucleotide *n*_2 _of sequence 2 and the sequences **x**_1 _and **x**_2 _are emitted (by the HMM). The computation of this joint probability is expressed in terms of recursions involving a *forward-variable *and a *backward-variable *(see Remark 7 in Appendix Section 8). In order to define these variables, denote by *S*_*m*_(*n*_1_, *n*_2_) the event that the state is *m *at the point when *n*_1 _nucleotides corresponding to the first sequence and *n*_2 _nucleotides corresponding to the second sequence have been emitted. Equivalently, this is the event that the path of the HMM process through the trellis visits the trellis node (*n*_1_, *n*_2_, *m*).

The forward-variable is then defined as the joint probability

(10) αm(n1,n2)=P(Sm(n1,n2),x11n1,x21n2),

i.e., the probability that the subsequence x11n1 of *n*_1 _nucleotides is emitted in the first sequence, the subsequence x21n2 of *n*_2 _nucleotides is emitted in the second sequence, and the state (of the alignment Markov process) is to *m*. The backward variable is defined as the conditional probability

(11) βm(n1,n2)=P(x1n1+1N1,x2n2+1N2|Sm(n1,n2)),

i.e., the probability that subsequences x1n1+1N1 and x2n2+1N2 are observed given that state is *m *when *n*_1 _and *n*_2 _nucleotides have been emitted in the first and second sequence, respectively. Here *N*_1 _and *N*_2 _represent the lengths of sequences **x**_1 _and **x**_2_, respectively.

From the Markov property of the hidden alignment process, the joint probability in (9) can be written as

(12) *P*(*n*_1 _↔ *n*_2_, **x**_1_, **x**_2_) = *α*_*ALN*_(*n*_1_, *n*_2_)*β*_*ALN*_(*n*_1_, *n*_2_)

The forward variable can be computed recursively by noting that each node in the trellis has three distinct incoming feasible edges corresponding to the three possible values for the immediately previous state (see Figure 8). Hence, the forward variable is obtained as

(13) αm(n1,n2)=∑m′∈MP(Sm′(n′1(n1,m),n′2(n2,m)),Sm(n1,n2),x11n1,x21n2)

where in accordance with (7) and (8),

(14) $${n^{\prime }}_{1}(n_{1},m)=\{\begin{array}{ll} n_{1} & \text{if }m=\text{INS}2\\ n_{1}-1 & \text{otherwise} \end{array}$$

and

(15) $${n^{\prime }}_{2}(n_{2},m)=\{\begin{array}{ll} n_{2} & \text{if }m=\text{INS}1\\ n_{2}-1 & \text{otherwise} \end{array}$$

Now using the HMM state transition probabilities and the symbol emission probabilities defined in Section 7.1, it can be seen that the forward variable expression in (13), yields the recursion formula,

(16) αALN(n1,n2)=∑m∈Mτ(m,ALN)γALN(x1n1,x2n2)P(Sm(n1−1,n2−1),x11n1−1,x21n2−1)=∑m∈Mτ(m,ALN)γALN(x1n1,x2n2)αm(n1−1,n2−1)αINS1(n1,n2)=∑m∈Mτ(m,INS1)γINS1(x1n1,⊓)P(Sm(n1−1,n2),x11n1−1,x21n2)=∑m∈Mτ(m,INS1)γINS1(x1n1,⊓)αm(n1−1,n2)αINS2(n1,n2)=∑m∈Mτ(m,INS2)γINS2(⊓,x2n2)P(Sm(n1,n2−1),x11n1,x21n2−1)=∑m∈Mτ(m,INS2)γINS2(⊓,x2n2)αm(n1,n2−1)

Following a similar procedure, recursions for the backward variable are obtained:

(17) βm(n1,n2)=τ(m,INS1)γINS1(x1n1+1,⊓)P(x1n1+2N1,x2n2+2N2|SINS1(n1+1,n2))+τ(m,ALN)γALN(x1n1+1,x2n2+1)P(x1n1+2N1,x2n2+2N2|SALN(n1+1,n2+1))+τ(m,INS2)γINS2(⊓,x2n2+1)P(x1n1+1N1,x2n2+2N2|SINS2(n1,n2+1))=τ(m,INS1)γINS1(x1n1+1,⊓)βINS1(n1+1,n2)+τ(m,ALN)γALN(x1n1+1,x2n2+1)βALN(n1+1,n2+1)+τ(m,INS2)γINS2(⊓,x2n2+1)βINS2(n1,n2+1)

The joint probability that the sequences are emitted in the denominator of (9) can also be obtained from the forward variable as,

(18) P(x1,x2)≡P(x11N1,x21N2)=∑m∈Mαm(N1,N2)

Using (12) and (18) in Equation (9) the posterior probability of co-incidence of two nucleotides can be obtained as,

(19) P(n1↔n2|x11N1,x21N2)∑m∈Mαm(n1,n2)βm(n1,n2)∑m∈Mαm(N1,N2)

### 7.2.3 Boundary Conditions

Boundary conditions are required to initiate the recursions in Equations (16) and (17). The forward variable recurses on the previous states and backward algorithm recurses on next states. As a result forward variable needs a boundary condition at the starting symbol pairs and backward algorithm needs the boundary condition at the ending pairs. Since the method is employed over ncRNAs rather than arbitrarily chosen RNA segments, special dummy symbol pairs are introduced at the beginning, i.e., nucleotide positions (0,0) and at the end, viz, nucleotide positions (*N*_1_*+ *1, *N*_2 _+ 1).

The beginning state is then specified by:

(20) $$\alpha_{m}(0,0)=\{\begin{array}{cc} 1 & m=\text{ALN}\\ 0 & \text{otherwise} \end{array}$$

Similarly the end state is specified by:

(21) $$\beta_{m}(N_{1}+1,N_{2}+1)=\{\begin{array}{cc} 1 & m=\text{ALN}\\ 0 & \text{otherwise} \end{array}$$

#### 7.3 Probabilistic Alignment Constraint Computation

As indicated in Section 3 the alignment constraints are computed by utilizing a threshold on the posterior probability of co-incidence. This process can be summarized as follows:

1. Using the boundary condition (20) and the recursions (16), calculate the forward variable *α*_*m*_(*n*_1_, *n*_2_) over 3D trellis ((*n*_1_, *n*_2_, *m*) ∈ [1 : *N*_1_] × [1 : *N*_2_] × *M*).

2. Using the boundary condition (21) and the recursions (17), calculate backward variable *β*_*m*_(*n*_1_, *n*_2_) over same trellis.

3. Calculate probability P(**x**_1_, **x**_2_) of emission of the observed sequences using (18).

4. Using (19), compute the alignment posterior probability P(*n*_1 _↔ *n*_2 _| **x**_1_, **x**_2_), for all possible nucleotide alignment positions, i.e. for (*n*_1_, *n*_2_) ∈ [1 : *N*_1_] × [1 : *N*_2_].

5. Determine a statistical confidence threshold *P*_thresh _such that pair-wise alignments with a probability of lower than *P*_thresh _may be considered improbable and therefore excluded (from the computations in subsequent Dynalign stage).

6. Determine the constraint set **C **of allowable nucleotide position alignments between the sequences by thresholding the posterior probability at the chosen statistical confidence level *P*_thresh_, i.e.

(22) **C **= {(*n*_1_, *n*_2_) | P(*n*_1 _↔ *n*_2 _| **x**_1_, **x**_2_) > *P*_thresh_}

Note that (22) is identical to equation (2) in Section 3.

The constraints on alignment are then imposed a priori in Dynalign by considering (in the dynamic programming step) only the pair-wise alignment positions in **C**.

#### 7.4 Model Parameter and Threshold Estimation

Seed alignments from the RFAM database [37], are utilized to estimate HMM parameters and thresholds required for the determination of alignment constraint sets. The process uses multiple sequence alignments for the 10 RNA families: 5S RNA, archaeal RNAseP, bacterial RNAseP classA, bacterial RNAseP classB, bacterial SRP, eukaryotic SRP, Group1 catalytic intron, Group2 catalytic intron, Nuclear RNAseP, and tRNA. Within each family the set of sequences available are randomized and half are utilized in the training process for determining the HMM parameters and the other half are used for testing and for establishing threshold probability values as will be described subsequently. The RFAM database includes multiple alignments and in order to obtain pairwise alignments, all possible (unique) pairwise sequence alignments implied by the multiple alignments of member sequences within a family are used.

In order to allow the model to better represent observed statistics, sequence pairs are grouped into a number of "bins" and HMM parameters are estimated for each bin. Bins are established based on estimates of percent nucleotide-identity – an approach that has been commonly used for several probabilistic models [11,36]. A brief description of the process follows. First, a "universal" set of HMM parameters is obtained by utilizing all alignments in the training set (from the 10 RNA families). Using this universal set of parameters, for any sequence pair, a maximum-likelihood (ML) alignment is performed which is then utilized to compute the percentage nucleotide-identity, i.e., the percentage of positions in the ML alignment that are aligned and have matching nucleotides. The seed alignments in the training set are then utilized to estimate the HMM model parameters, i.e., the state-transition and emission probabilities, for sequence pairs with percent nucleotide identities corresponding to the bins (see Remark 8 in Appendix Section 8): < 30%, 30 – 40%,40 – 50%,50 – 60%, 60 – 70%, 70 – 80%, 80 – 90%, and 90 – 100%. Once the HMM parameters are known, for any pair of sequences, first using the ML alignment with the universal model, the percent nucleotide identity is computed which is then used to identify the corresponding bin for the HMM parameters. Using these parameters, the posterior probability of nucleotide co-incidence may be calculated as described in Section 7.2.

**Figure 9:**
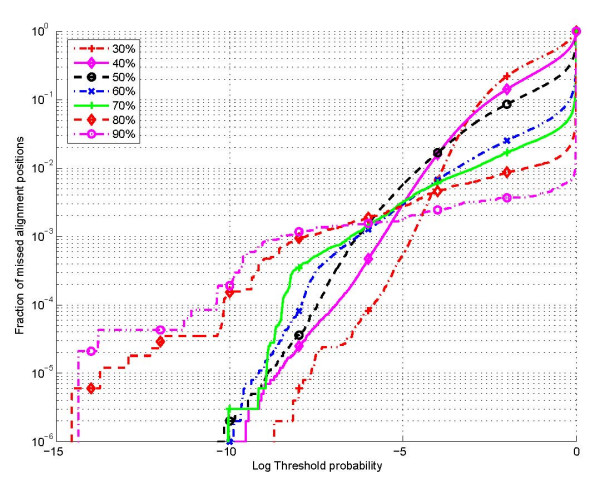
Fraction of aligned base pairs missed as a function of probability threshold for restricting alignments.

**Figure 10:**
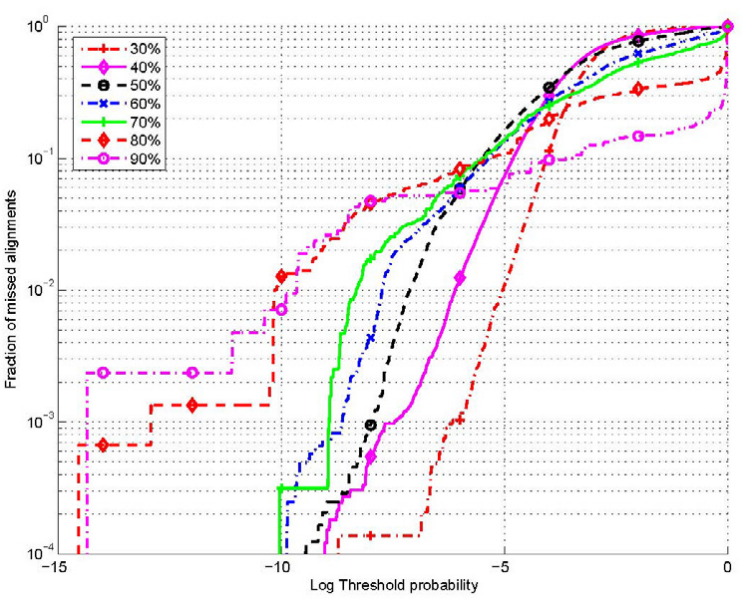
Fraction of sequence alignments missed as a function of probability threshold for restricting alignments.

Once the HMM parameters are established, suitable values of probability thresholds (*P*_thresh _in (2)), are determined by empirically evaluating the performance of the alignment constraint set over test sequences (not included in the training). Since nucleotide alignments outside the alignment constraint set are disallowed, it is desirable that as few as possible of the true nucleotide alignment positions are outside the constraint sets. Figure 9 shows a plot of the fraction of missed alignment position pairs as a function of the (logarithm of) threshold *P*_thresh _for the 7 different bins indicated above (over 5s and tRNA sequences in the test set). Figure 10 illustrates the fraction of missed alignments, i.e., alignments for which even one nucleotide alignment position is outside the corresponding constraint set. Using Figure 9, values of the threshold probability (*P*_thresh_) were determined for each of the 7 bins that ensured that less than one in 10,000 alignment positions were missed, i.e., the empirical probability of missing an alignment position was less than 10^-4^. These threshold values were then utilized in subsequent experiments to determine alignment constraints for Dynalign.

#### 7.5 Scoring Structure Predictions

The accuracy in correctly predicting canonical base pairings is utilized for the purpose of scoring the performance of the structural prediction methods. The performance is quantified using *sensitivity*, i.e., the fraction of canonical pairings in the known (or true) structure that are correctly predicted by the method and the *positive predictive value *(PPV), which is defined as the fraction of predicted base pairings that are in agreement with the known structure. In both cases, a "slippage" of one nucleotide on one side of the base pairing is allowed. Thus a base pairing *i *- *j *in the known structure is considered correctly predicted (for computation of sensitivity) if the predicted structure contains a base pairing in one of the 5 positions: *i *- *j*, (*i *± 1) - *j*, *i *- (*j *± 1). Likewise, a base pairing *i *- *j *in the predicted structure is considered correctly predicted (for computation of PPV) if the known structure contains a base pairing in one of the *5 *positions: *i *- *j*, (*i *± 1) - *j*, *i *- (*j *± 1).

Thus,

(23) $$\text{Sensitivity}=\frac{N_{c}^{k}}{N_{t}^{k}}$$

where $N_{c}^{k}$ is the number of base pairings in the known (k) structure that are correctly (c) predicted as per the above definition, and $N_{t}^{k}$ is the total (t) number of base pairings in the known structure.

Similarly,

(24) $$\text{PPV}=\frac{N_{c}^{p}}{N_{t}^{p}}$$

where $N_{c}^{p}$ is the number of base pairings in the predicted (p) structure that are correct (c) as per the above definition, and $N_{t}^{p}$ is the total (t) number of base pairings in the predicted structure.

The motivation for allowing the one nt slippage is twofold: firstly, the slippage of one nt does not typically change the predicted topology of the secondary structure, which is much more significant than the exact pairing predicted. Secondly, the "correct" pairings are obtained using experiments and comparative sequence analysis, which also have uncertainty due to dynamics of base pairings and limited resolution of the methods. The scoring methodology based on the one nt slippage has also been used in prior published literature [6,24,48]. The scoring method adopted yields values of sensitivity and PPV roughly 2–3% higher than when an exact match criterion is used for scoring, where a base pairing *i *- *j *in the known structure is deemed correctly predicted for the purpose of sensitivity computation if and only if the *exact *base pairing *i *- *j *is also predicted (and likewise exact match is used for PPV computation). For completeness, sensitivity and PPV values obtained with the exact match constraints for scoring are included as Tables 10 and 11.

**Table 10:** Structural prediction accuracy statistics for the methods benchmarked over 2000 random tRNA selections when scored using an exact match criterion.

		Percent sequence similarity				
		
		20–40	40–60	60–80	80–100	0–100
**Dynalign new constraint**	Sens	0.818	0.862	0.923	0.715	0.861
	PPV	0.788	0.832	0.916	0.697	0.834
**Dynalign *M *constraint**	Sens	0.813	0.854	0.917	0.716	0.855
	PPV	0.781	0.822	0.907	0.698	0.825
**FOLDALIGN**	Sens	0.731	0.846	0.887	0.690	0.835
	PPV	0.815	0.894	0.939	0.846	0.889
**StemLoc**	Sens	0.581	0.894	0.940	0.872	0.860
	PPV	0.747	0.899	0.924	0.876	0.886
**Consan**	Sens	0,786	0.905	0.944	0.769	0.894
	PPV	0.755	0.848	0.873	0.667	0.838
**Single Prediction**	Sens	0.743	0.746	0.774	0.717	0.748
	PPV	0.696	0.688	0.728	0.673	0.693

Results are summarized for sequence similarity values ranging from 20% through 100% in steps of 20% and for the overall data set (0 – 100). Dynalign new constraint refers to Dynalign with probabilistic alignment constraints proposed here. Software version numbers and parameters for the algorithms are described in Section 7.6.

**Table 11:** Structural prediction accuracy statistics for the methods benchmarked over 2000 random 5S RNA selections when scored using an exact match criterion.

		Percent sequence similarity				
		
			40–60	60–80	80–100	0–100
**Dynalign new constraint**	Sens	0.850	0.870	0.876	0.869	0.871
	PPV	0.790	0.791	0.782	0.743	0.785
**Dynalign *M *constraint**	Sens	0.847	0.869	0.876	0.869	0.870
	PPV	0.789	0.789	0.778	0.744	0.782
**FOLDALIGN**	Sens	0.684	0.721	0.772	0.501	0.724
	PPV	0.724	0.750	0.789	0.562	0.752
**StemLoc**	Sens	0.253	0.631	0.858	0.711	0.698
	PPV	0.603	0.751	0.854	0.738	0.786
**Consan**	Sens	0.602	0.743	0.882	0.759	0.786
	PPV	0.598	0.704	0.768	0.608	0.717
**Single Prediction**	Sens	0.655	0.695	0.734	0.744	0.709
	PPV	0.579	0.606	0.641	0.632	0.618

Results are summarized for sequence similarity values ranging from 20% through 100% in steps of 20% and for the overall data set (0 – 100). Dynalign new constraint refers to Dynalign with probabilistic alignment constraints proposed here. Software version numbers and parameters for the algorithms are described in Section 7.6.

#### 7.6 Parameters for Dynalign and Other Programs used for Benchmarking

For all the experiments, Dynalign is run with constraints on folding where base pairs whose minimum free energy structure is above 70% of minimum free energy structure as determined by single sequence secondary structure prediction are not allowed to pair [35]. This is the default setting and is used in both Dynalign with *M *constraints and Dynalign with probabilistic alignment constraints. In addition, Dynalign with the *M *constraint uses *M *= 7 as explained in [35]. Only the minimum free energy structures were predicted, i.e. suboptimal foldings are not generated. The thermodynamic parameters used in both versions of Dynalign are those compiled in references [19,25]. Dynalign with *M *constraint used all parameters identical to the ones for the new method with the exception of the alignment constraint, which was replaced instead by the banded constraint implied by *M *and defined in Equation (1).

For the purposes of benchmarking, the new proposed method was compared against single structure prediction [19] and four other pairwise structural prediction methods: Dynalign with the previous *M *constraint [35], StemLoc [11], Consan [34], and FOLDALIGN [33]. These programs were utilized in their default configuration. A summary description of these methods and the runtime options utilized in the benchmarking experiments follows:

• *StemLoc*: *StemLoc *[11]*is a pairwise structural alignment prediction program based on stochastic context-free grammars. It uses "fold" and "alignment" envelopes to reduce computation and memory. The benchmarking experiments utilized StemLoc version 0.19b in global alignment mode ('-g' option) with 100 best alignments ('-na 100' option) and 1000 ('-nf 1000' option) best folds*.

• Consan: Consan [34] is a pair-SCFG for making pairwise structural alignment. It utilizes the concept of "pins", i.e. nucleotide positions that are constrained to be aligned, in order to constrain the alignment space and thereby limit computation and memory. Pins are selected based on posterior probabilities of alignment. The benchmarking was performed using Consan version 1.2. The training required for Consan was performed over the dataset of LSU and SSU RNAs included with the package (using 'mltrain -s mixed80.mod mixed80.stk'). The resulting model file "mixed80.mod" is used for Consan runs (using 'sfold -S -m mixed80.mod fastal fasta2'). The '-S' option is used for suppressing all messages to standard output except the structural information that Consan predicts.

• *FOLDALIGN: FOLDALIGN *[33]*is free energy minimization based Sankoff implementation for local structural alignment of multiple sequences. The benchmarks were computed using FOLDALIGN version 2.0.3 in global alignment mode ('-global' option) since the input consisted of homologous tRNA or 5S RNA pairs*.

• Single Prediction: The single sequence prediction method is based on thermodynamic free energy minimization [49]. Our implementation utilized the version included in the current version of RNA Structure and Dynalign [19].

Dynalign and the single sequence prediction method can utilize knowledge of modified nucleotides that cannot accommodate the canonical A-form helix (communicated in the form of lower case alphabets for the corresponding bases in the input). These are forced single-stranded in the structural prediction and the knowledge therefore improves the overall prediction [24]. The tRNA database includes knowledge of modified nucleotides whereas the 5S RNA database does not include any knowledge of modified nucleotides. Since the other methods benchmarked do not utilize knowledge of modified nucleotides, for the benchmarking results of Section 4.4, the knowledge of modified nucleotides was *not utilized *for the Dynalign and single sequence predictions. Results in Section 4 list the performance both with and without the use of this knowledge in prediction.

Computation times for all the methods are determined by using the *time *command under Linux. Memory requirements for the programs are as reported in the *size *entry of *Linux ps *command. For the Dynalign methods the memory usage is estimated after all requisite dynamic allocations are done. For other programs, the memory usage is estimated at exactly half of the run time for each sequence pair, except for sequences with computation times over 120 seconds, for which the memory usage is evaluated at 60 seconds.

## Authors' contributions

G.S. proposed the use of *a posteriori *probability estimates for the purpose of determining alignment constraints. All three authors worked together on the conceptual development of the technique. A.O.H. implemented the proposed algorithm and conducted the experiments for obtaining the reported results. In this effort he was guided and supervised by G.S. and D.H.M. D.H.M. also provided assistance in the integration of the software with existing Dynalign code. The three authors collectively wrote, edited, and revised the manuscript. Significant connections to related work in the biological and bioinformatics literature were recognized by D.H.M. and in electrical communications literature by G.S., respectively. All authors read and approved the final manuscript.

## 8 Appendix

1. Note that the set of aligned positions alone (black blocks) does not satisfy this requirement.

2. The actual search is 4-dimensional, which can be thought of as combination of a two-dimensional search over possible alignments between the sequences along with a two-dimensional search over (identical) folds for the sequences.

3. The prediction accuracy for Dynalign also depends on the thermodynamic model employed for scoring secondary structures and hence the dependence on the alignment constraint is indirect.

4. A reasonably high value of specificity is also advantageous because it reduces computation time and memory.

5. The assumption of time invariance may be dropped if necessary. It is adopted for notational simplicity.

6. Some of the details of the trellis representation are a matter of convention and there exist other valid conventions that may be adopted instead.

7. In computational biology literature, the terms forward matrix and backward matrix are alternately used for the forward and backward variables.

8. Note that random nucleotide sequences of the same length can be expected to have a sequence similarity of 25%.
